# Impact of health insurance status among migrants from sub-Saharan Africa on access to health care and HIV testing in Germany: a participatory cross-sectional survey

**DOI:** 10.1186/s12914-019-0189-3

**Published:** 2019-03-05

**Authors:** Johanna Müllerschön, Carmen Koschollek, Claudia Santos-Hövener, Anna Kuehne, Jacqueline Müller-Nordhorn, Viviane Bremer

**Affiliations:** 1 University of Applied Sciences Fulda, Leipzigerstr. 123, 36037 Fulda, Germany; 2 Department for Infectious Disease Epidemiology, Division for HIV/AIDS, STI and Blood-borne Infections, Robert Koch Institute, Seestr. 10, 13353 Berlin, Germany; 3 Charité – Universitätsmedizin Berlin, Berlin, Germany; 4 European Programme for Intervention Epidemiology Training (EPIET), ECDC, Granits väg 8, 171 65 Solna, Stockholm, Sweden; 5 Institute of Public Health, Charité – Universitätsmedizin Berlin, Seestr. 73, 13347 Berlin, Germany

**Keywords:** Transients and migrants, Human migration, Health insurance, Access to health care, Health care utilization, HIV

## Abstract

**Background:**

Among all newly diagnosed HIV cases in Germany in 2015, 16% originated from sub-Saharan Africa. Twelve percent of these infections were contracted within Germany and migrants from sub-Saharan Africa (misSA) are diagnosed later than Germans. Migrants, specifically those without health insurance, face many barriers accessing health care due to their residence status and cultural, socio-economic, legal and linguistic barriers. We assessed whether misSAs’ access to healthcare and utilization of HIV testing services depends on their health insurance status to inform prevention strategies.

**Methods:**

From January 2015 to February 2016, we conducted a cross-sectional survey on knowledge, attitude, behavior, practice (KABP) regarding HIV, viral hepatitis and sexually transmitted infections among misSA in Germany. The survey was a community-based participatory research project; trained peer researchers recruited participants through outreach. To detect differences between participants with a regular health insurance card compared to asylum seekers with a medical treatment voucher or participants without health insurance or medical treatment voucher, unadjusted and adjusted Odds Ratios, chi-squared tests and 95% confidence intervals were calculated.

**Results:**

A total of 1919 cases were considered. Overall, 83% had a health insurance card, 10% had a medical treatment voucher and 6% had no health insurance. Participants living in Germany for less than 5 years were less likely to have a health insurance card and more likely to have lower German language skills. Participants without health insurance visited a physician in case of health problems less often than participants with medical treatment voucher or a health insurance card (41.2% vs. 66.1% vs. 90%). Participants without health insurance reported less frequently visiting physicians or hospitals and were less likely to undergo a HIV test.

**Conclusion:**

Having no health insurance or medical treatment voucher decreased the odds of contact with the healthcare system more than other socio-demographic characteristics. Furthermore, misSA without health insurance had lower odds of ever having done an HIV test than participants with health insurance. To increase health care utilization and testing and to ensure adequate medical care, all migrants should get access to health insurance without increasing costs and consequences for residence status.

## Background

### Access to healthcare for asylum seekers and migrants in Germany

The large majority (> 95%) of the population in Germany holds private or statutory health insurance [1] and therefore a health insurance card (HIC). Patients without a health insurance card (HIC) have difficulties accessing health care, unless they can afford to cover the bills by themselves [2]. There are 80,000 people living in Germany without health insurance [1] despite there being compulsory health insurance (Sozialgesetzbuch §5) [3]. Those mainly affected by the absence of health insurance are self-employed, migrants, homeless people and people without legal residence status [2, 4]. The German Asylum Law (§4 and §6) [Asylberwerberleistungsgesetz] regulates access to health care for asylum seekers and undocumented migrants. Access to medical care is offered mainly for acute diseases and obstetric conditions, but not for preventive medical check-ups. In many federal states, asylum seekers have to obtain a medical treatment voucher (MTV) from social welfare offices each time they want to seek medical care, thus decisions about whether the treatment is necessary are not made by professional medical staff. Due to § 2 of German Asylum Law [Asylberwerberleistungsgesetz] [5] migrants in Germany have the right to access the regular health care system after 15 months of stay.

According to §87 of the German Residence Act [Aufenthaltsgesetz], public institutions have to report migrants without a valid residence permit to the foreigners’ registration offices, including social welfare offices which grant MTV. Medical staff and administration of medical institutions are excluded from this regulation, however, reporting still occurs. In addition, the administration is concerned about treatment costs [6]. If a person cannot cover the bill for treatment on his/her own, the corresponding bill will be sent along with their personal data to the social welfare office. Thus, migrants without a residence permit risk being deported when seeking medical care if they are not able to pay for it.

Several studies show that access to healthcare is limited for migrants, due to language barriers, lower educational levels and a lack of multicultural services [7, 8]. Those particularly affected are migrants without health insurance, for example recently arrived migrants who need medical care as well as those without any form of residence status [9, 10]. Free medical care services outside of the regular system are mainly available in bigger cities, for example offices for medical assistance for refugees (Medibueros) [11].

In 2015 almost 220,000 migrants from sub-Saharan Africa (misSA) were officially residing in Germany according to statistics from the foreigners’ registration offices [12]. This does not include misSA who do not have a legal residence status or misSA who have a German citizenship [12].

### Epidemiological situation and prevention of HIV in Germany

Since 2009, the number of diagnoses for heterosexual transmission of HIV has been increasing among newly diagnosed HIV cases in Germany [13]. In 2015, among all newly diagnosed cases with heterosexual transmission, 59% were misSA and 16% of all people diagnosed with HIV in Germany originated from sub-Saharan Africa. Approximately 12% of these infections were contracted in Germany. In addition, HIV diagnoses among misSA were more frequently discovered in later clinical stages compared to other populations [13]. This might indicate difficulties in accessing healthcare, HIV prevention and testing services.

In Germany, testing for HIV can be conducted by private practitioners and hospitals as well as local public health departments and non-governmental organizations (NGOs). The utilization of these services is lower in migrant populations [2, 14]. Studies have shown that migrants have an increased vulnerability for HIV as access to prevention, counseling and testing is limited due to cultural, social, legal and language barriers [14, 15]. Stigma, discrimination, residence issues and traumatic experiences associated with the migration process can also present barriers in accessing healthcare services and preventive measures. Moreover, a lack of culturally sensitive services and language/translation capacities to reach different migrant groups, as well as migrants’ lack of knowledge about existing services, might present important barriers to HIV prevention [2, 9, 16].

For the successful implementation of HIV and STI prevention, the World Health Organization (WHO) and European Centre for Disease Prevention and Control (ECDC) recommend combining routine biological surveillance as well as the monitoring of behavioral indicators on HIV, viral hepatitis (HEP) and sexually transmitted infections (STI) in migrant populations, to identify knowledge gaps and behavioral risk factors [17, 18]. To date in Germany there have been no specific studies conducted at a national level investigating knowledge, attitudes, behavior, practice (KABP) and needs of misSA towards the utilization of healthcare services in general and especially for HIV, HEP and STI.

In 2015–16 we conducted a cross-sectional KABP survey on HIV, HEP and STI among misSA in six cities in Germany. The overall aim of this study was to identify gaps in knowledge and behavioral patterns that need to be addressed with future prevention measures. This work focuses on misSAs’ access to healthcare and utilization of HIV testing services depending on their health insurance status, ranging from no health insurance at all (NI), MTV and HIC. We analyzed a subset of data from four of six study cities (Munich, Essen, Cologne and Berlin (Munich, Essen, Cologne and Berlin; data collection in Frankfurt and Hanover was not yet finished) to identify barriers of access to health care and on the uptake of HIV testing, to demand political action and access to health insurance for everybody.

## Objective

The aim of this paper is to identify which factors influence the health insurance status of misSA and the impact of health insurance status on the utilization of the German healthcare system. Furthermore, we investigated the impact of the health insurance status on the uptake of HIV testing.

## Methodology

### Study design and questionnaire

In 2011 the Robert Koch Institute (RKI) started a research process in cooperation with the German AIDS Service Organization (Deutsche Aids-Hilfe, DAH), the Federal Centre for Health Education (Bundeszentrale für gesundheitliche Aufklärung, BZgA) and African communities. The study was planned as a community-based participatory research project [19]. Representatives from various African communities, practitioners from HIV and STI prevention, experts in the field of migration research, experts in HIV/STI testing and STI surveillance collaboratively formed the aims, objectives and methodology for the study.

The questionnaire was developed by an expert group consisting of representatives from HIV/STI clinics and specialists, misSA community members and researchers [20]. Trained community members conducted cognitive testing of the questionnaire with five misSA. The questionnaire was pre-tested with 35 community members and a pilot study in Hamburg was conducted subsequently [21]. The feedback from the pilot study in Hamburg was used to adapt and pretest the questionnaire again before implementing it in this study [20].

Through a standardized paper-based anonymous questionnaire, socio-demographic information, information on knowledge, attitude and behavior was recorded regarding HIV, HEP, STI, testing, preventive care and medical care [20]. The questionnaire determined knowledge gaps (e.g., Did you know this before now? AIDS is caused by a virus called HIV.). Further questions asked were based on recommended behavior surveillance indicators for migrant populations by ECDC [22]. Possible modes of administration were self-completion, face-to-face or telephone interview. The questionnaires were offered in English, French and German and given out with a postpaid envelope to RKI. In addition, all peer researchers were proficient in several African languages.

Specific instructions on the development of the study design and questionnaire, the pilot study in Hamburg and the methodology have been described elsewhere [20, 21, 23].

Between January 2015 and February 2016, we conducted the survey among misSA living in Germany in six German cities and regions (Munich, Cologne and Essen (Rhine-Ruhr-Region) Berlin, Frankfurt and Hanover), using a convenience sample, random sampling was not feasible for logistical reasons. The study was conducted in German cities with > 2000 misSA residents according to statistics of foreigners’ registration offices [12]. The aim was to recruit a minimum of 2550 misSA in six German cities. Differences in proportions of 10% (45% vs. 55%) between men and women should be detectable with a significance level of .05 and accepting a beta error of .2. To report results to the local partner organizations, a minimum of 350 misSA per city was decided. Study participants were recruited through trained African peer researchers, who had access to their local communities. More details on the sample size calculation and on the recruitment process are published elsewhere [21].

Findings were discussed and evaluated in focus group discussions with misSA [20, 21]. After data collection and analysis, a meeting with local policy makers, stakeholders and community partners was conducted to present results and to collaboratively formulate recommendations for local prevention planning.

### Definitions

African regions were categorized following the German Federal Statistical Office [Statistisches Bundesamt Deutschland [12]].

Access to health care includes many more factors than health insurance status such as financial, linguistic, communicational, socio-cultural, structural, political barriers [2, 4, 7, 9, 10]. In this survey, health insurance status is a surrogate marker for official or political restrictions when seeking health care depending on the insurance status. In our study, access was defined by the possibility to access health care. Utilization means that somebody accesses care such as visiting a physician or hospital. Utilization was seen as a consequence of health needs and access to healthcare.

To measure differences in access and utilization of healthcare, we focused on health insurance status. We compared three groups: 1. individuals with a health insurance card (HIC), 2. individuals with a medical treatment voucher (MTV) and 3. individuals with no health insurance at all (NI).

Table 1 shows the operationalization of the primary outcome health insurance status and of the secondary outcomes utilization of medical care and the uptake of HIV testing.

**Table 1:** Outcomes

	Assessment
Primary outcome	
Health insurance status	Comparing HIC vs. MTV and HIC vs. NI
Secondary outcomes	
Utilization of medical care	Physician or hospital visit within the last year yes/no
Uptake of HIV testing	HIV ever been tested yes/no

### Statistical analysis

We used Voxco Interviewer Web™ (an online survey and data collection software) for data entry and imported the dataset to IBM SPSS Statistics 20.0 for data cleaning. Reasons for excluding questionnaires were if interviewees were not living in Germany, if they were younger than 18 years, if their sex was not stated, if ≤60% of the questionnaire were filled in completely, or neither participants nor one of their parents originated from sub-Saharan Africa.

To describe the study population, we used frequency tables and measurements of central tendencies. We used bivariate analysis to determine potential differences in characteristics or groups. Participants with missing information on health insurance status were excluded from the bivariate analysis. We calculated chi-squared tests, odds ratios (OR) and 95% confidence intervals to detect associations. We stratified by socio-demographic variables to determine associations and to identify interactions. For descriptive analysis, we stratified by health insurance status as defined above.

We conducted multivariate analysis (MVA) using logistic regression to determine the association between health insurance status, the last consultation to a physician or hospital within the last 12 months and the uptake of HIV testing. We adjusted for the following confounders: sex (categorical, 2 groups), age (categorical, 4 age groups) and administration mode (categorical, 3 groups). Also all socio-demographic factors showing a significance level of *p* ≤ 0.05 in univariate analysis, were included into the MVA (time living in Germany (categorical, 5 time groups), level of German language (categorical, 6 level groups), religion (categorical, 3 groups (Christians, Muslims and other or no religious)), income (categorical, 5 income groups), region of birth (categorical, 4 birth regions) and school education (categorical, 5 groups)). We also adjusted for mode of survey administration in the MVA when it had shown to be significantly associated with the outcome in univariate analysis. Participants who did not know their insurance status were excluded from MVA.

We controlled for interactions between time living in Germany, health insurance status, education and German language skills. The statistical analysis was conducted using IBM SPSS Statistics 20.0.

## Results

### Study population

In total 2089 valid questionnaires were received from the cities of Munich, Essen, Cologne and Berlin. A total of 170 cases (8.1%) were excluded from further data analysis. Reasons for the exclusion were: interviewees were not living in Germany (*n* = 7), younger than 18 years (*n* = 15), sex was not stated (*n* = 25), or ≤ 60% of the questions were completed (*n* = 29) or neither participants nor one of their parents originated from sub-Saharan Africa (*n* = 11) or reported to live in Germany since their birth (*n* = 83). After exclusion, 1919 valid cases were included in the following analysis.

### Demographic characteristics

In total, 850 (44%) participants were female and 1069 (56%) male. The median age was 33 years. The majority of participants were born in Western Africa (56%), followed by Central Africa (25%), Eastern Africa 14%, and Southern Africa (5.6%).

Median time of duration of residence in Germany was 7 years. More than a third of participants reported living in Germany for less than five years (41%). A total of 34% of participants had a university degree, 33% had finished high school or vocational school and 25% had a primary or secondary school degree. The majority of the study population was Christian (70%), 24% were Muslim and 5.2% reported to follow no religion (for further socio-demographic information see Table 2).

**Table 2:** Health insurance status and socio-demographic characteristics of the study population

Insurance status	Health insurance card	Medical treatment voucher	No health insurance	I do not know	*p*-value
Age group	*n* (%)	*n* (%)	*n* (%)	*n* (%)	
18–25 years	224 (67.3%)	63 (18.9%)	34 (10.2%)	12 (3.6%)	*p* < 0.001
26–35 years	490 (79.8%)	82 (13.4%)	35 (5.7%)	7 (1.1%)	
36–45 years	392 (89.3%)	21 (4.8%)	21 (4.8%)	5 (1.1%)	
≥ 46 years	336 (93.9%)	4 (1.1%)	16 (4.5%)	2 (0.6%)	
Level of education					
Primary/ Secondary School	328 (70.1%)	87 (18.6%)	40 (8.5%)	13 (2.8%)	*p* < 0.001
High School/ Vocational School	541 (87.4%)	52 (8.4%)	21 (3.4%)	5 (0.8%)	
University/ College	587 (90.7%)	11 (1.7%)	44 (6.8%)	5 (0.8%)	
No certificate	93 (73.8%)	22 (17.5%)	8 (6.3%)	3 (2.4%)	
Other	11 (64.7%)	4 (23.5%)	1 (5.9%)	1 (5.9%)	
Level of German language					
Mother tongue	49 (95.1%)	1 (2.4%)	1 (2.4%)	0 (0.0%)	*p* < 0.001
Very good	421 (96.6%)	4 (0.9%)	10 (2.3%)	1 (0.2%)	
Good	468 (94.9%)	14 (2.8%)	8 (1.6%)	3 (0.6%)	
Satisfactory	358 (86.7%)	38 (9.2%)	13 (3.1%)	4 (1.0%)	
Little	228 (66.3%)	72 (20.9%)	37 (10.8%)	7 (2.0%)	
None	43 (28.9%)	50 (33.6%)	44 (29.5%)	12 (8.1%)	
Religion					
Christians	1113 (85.0%)	108 (8.2%)	77 (5.9%)	12 (0.9%)	*p* = 0.001
Muslim	344 (77.0%)	58 (13.0%)	33 (7.4%)	12 (2.7%)	
No or other religion	97 (85.1%)	10 (8.8%)	4 (3.5%)	3 (2.6%)	
Monthly NET income					
I do not want to answer	305 (84.5%)	23 (6.4%)	27 (7.5%)	6 (1.7%)	*p* < 0.001
< 500€	161 (66.0%)	64 (26.2%)	14 (5.7%)	5 (2.0%)	
500 – < 1000 €	333 (95.1%)	9 (2.6%)	7 (2.0%)	1 (0.3%)	
1000 – < 2000 €	433 (98.2%)	3 (0.7%)	4 (0.9%)	1 (0.2%)	
≥ 2000 €	111 (94.9%)	2 (1.7%)	4 (3.4%)	0 (0.0%)	
No income	157 (58.1%)	52 (19.3%)	50 (18.5%)	11 (4.1%)	
Region of birth					
Western Africa	815 (80.5%)	109 (10.8%)	69 (6.8%)	20 (2.0%)	*p* = 0.051
Central Africa	389 (86.3%)	39 (8.6%)	22 (4.9%)	1 (0.2%)	
Eastern Africa	209 (83.9%)	24 (9.6%)	10 (4.0%)	6 (2.4%)	
Southern Africa	94 (90.4%)	4 (3.8%)	6 (5.8%)	0 (0.0%)	

### Access to health care

#### Factors associated with health insurance status

Overall, 83% (*n* = 1556) of participants had HIC, 10% (*n* = 180) required MTV, 6.0% (*n* = 114) reported to have NI and 1% (*n* = 27) did not know their health insurance status.

Duration of residence in Germany and German language skills were associated with health insurance status. With increasing time living in Germany, the proportion of participants having MTV or NI decreased (Table 3). Among the participants that required MTV, 89% were living in Germany for less than five years. Furthermore, 83% of participants with NI were living in Germany for less than five years. MisSA living in Germany for less than one year had HIC or MTV less often. Still, 16 participants living in Germany for more than ten years required MTV or had NI.

**Table 3:** Duration of residence in Germany and health insurance status (*n* = 1854), *p* < 0.001

Duration of residence	Under one year	1- under 5 years	5- under 10 years	10- under 20 years	20 years and more	Total
Insurance status	*n* (%)	*n* (%)	*n* (%)	*n* (%)	*n* (%)	*n* (%)
Health insurance card	79 (36.1%)	411 (73.4%)	324 (93.4%)	478 (98.0%)	261 (97.4%)	1543 (83.2%)
Medical treatment voucher	59 (30.9%)	101 (18.0%)	10 (2.9%)	5 (1.0%)	4 (1.5%)	179 (9.7%)
No insurance	49 (25.7%)	38 (6.8%)	11 (3.2%)	4 (0.8%)	3 (1.1%)	105 (5.7%)
I do not know	14 (7.3%)	10 (1.8%)	2 (0.6%)	1 (0.2%)	0 (0.0%)	27 (1.5%)

In comparison HIC and MTV vs. NI, participants with very good or good German language skills had higher odds of being integrated within the regular insurance system (HIC and MTV) than those with intermediate to no German language skills (96% vs. 69%; OR = 9.70, 95% CI: 6.93–13.67).

MVA revealed the influence of duration of residence in Germany and German language skills on health insurance status (to have a HIC vs. MTV (Model A) or HIC vs. NI (Model B)). As shown in Table 4, higher educational levels were associated with higher likelihoods of having HIC in comparison to MTV or NI. In both models, having no income was negatively associated with having HIC. Sex, age, religion and region of birth were not associated with health insurance status.

**Table 4:** Multivariate analysis on health insurance status (including factors significantly associated with health insurance status in univariate analysis)

Variable	Model A: HIC (1) vs. MTV (0)			Model B: HIC (1) vs. NI (0)		
	aOR	95% - CI	*p*-value	aOR	95% - CI	*p*-value
Sex						
Men	Ref.			Ref.		
Women	1.55	0.96–2.51	0.075	1.63	0.92–2.89	0.092
Age group						
18–25 years	1.44	0.81–2.55	0.215	1.41	0.68–2.93	0.361
26–35 years	Ref.			Ref.		
36–45 years	1.44	0.69–3.01	0.333	0.62	0.30–1.28	0.194
≥ 46 years	3.93	1.14–13.57	0.300	0.49	0.19–1.22	0.126
Length of stay in Germany						
≤ 1 year	**0.10**	**0.03–0.29**	**< 0.001**	**0.19**	**0.07–0.51**	**0.001**
1 - < 5 years	**0.19**	**0.08–0.49**	**0.001**	0.69	0.29–1.64	0.405
5 - < 10 years	Ref.			Ref.		
10 - < 20 years	0.96	0.25–3.72	0.957	**3.82**	**1.10–13.22**	**0.035**
≥ 20 years	0.50	0.11–2.39	0.388	4.04	0.89–18.34	0.105
German language skills						
None	**0.12**	**0.04–0.32**	**< 0.001**	**0.12**	**0.04–0.34**	**< 0.001**
Little	**0.19**	**0.08–0.44**	**< 0.001**	**0.30**	**0.12–0.76**	**0.011**
Satisfactory	**0.37**	**0.16–0.87**	**0.022**	1.24	0.41–3.76	0.700
Good	Ref.			Ref.		
Very good	1.00	0.28–3.60	0.998	0.56	0.19–1.63	0.288
Mother tongue	0.00	< 0.000	0.998	0.25	0.03–2.34	0.225
Religion						
Christian	Ref.			Ref.		
Muslim	1.22	0.69–2.16	0.497	1.25	0.64–2.47	0.517
No or other religion	1.90	0.60–5.98	0.272	0.96	0.25–3.66	0.946
Education						
Other	0.55	0.10–2.99	0.491	–	–	–
No degree or certificate	1.50	0.72–3.14	0.284	2.38 00.54	0.76–7.52	0.138
Primary or secondary school	Ref.			Ref.		
High −/vocational school	1.39	0.79–2.42	0.252	**2.45**	**1.10–5.47**	**0.028**
University degree	**15.41**	**6.67–35.57**	**< 0.001**	1.44	0.73–2.82	0.289
Monthly NET income						
I do not want to answer	0.49	0.20–1.20	0.119	**0.32**	**0.12–0.83**	**0.019**
No income	**0.25**	**0.11–0.58**	**0.001**	**0.23**	**0.09–0.58**	**0.002**
< 500 €	**0.14**	**0.06–0.33**	**< 0.001**	0.37	0.13–1.03	0.057
500 € - < 1.000 €	Ref.			Ref.		
1.000 € - < 2.000 €	6.32	0.75–53.04	0.090	1.72	0.46–6.45	0.423
≥ 2.000 €	0.40	0.07–2.34	0.307	0.50	0.12–2.10	0.343
Region of birth						
Western Africa	Ref.			Ref.		
Central Africa	1.08	0.56–2.06	0.828	0.90	0.45–1.78	0.753
Eastern Africa	0.74	0.35–1.57	0.430	1.62	0.66–4.02	0.295
Southern Africa	0.98	0.27–3.56	0.972	1.09	0.33–3.57	0.885

Significant results are marked bold

### First contact point in case of health problems

The majority of participants (84%) reported visiting a physician, followed by hospitals (31%) or pharmacies (20%) (multiple answers were possible) when experiencing medical problems. Only 26 (1.4%) participants reported not knowing where to go in case of health issues. The majority (23/88%) of these participants did not have a HIC. Participants with a HIC were more likely to visit the physician (90% vs. 66% vs. 41%; *p* < 0.001) or pharmacy (23% vs. 4% vs. 17%; *p* < 0.001) than participants with MTV or NI. Participants with HIC less often reported asking friends (6% vs. 12% vs. 18%; *p* < 0.001) or not knowing where to go in case of health issues (0.2% vs. 1% vs. 16%; *p* < 0.001) (Table 5).

**Table 5:** Participants by health insurance status and first contact point in case of health-related issues (*n* = 1884)

Insurance status	Health insurance card	Medical treatment voucher	No insurance	I do not know	Total	*p*-value
Contact point	*n* (%)	*n* (%)	*n* (%)	*n* (%)	*n* (%)	
Physician	1406 (90.0%)	119 (66.1%)	47 (41.2%)	17 (63.0%)	1589 (84.3%)	< 0.001
Hospital	494 (31.6%)	61 (33.9%)	23 (20.2%)	5 (18.5%)	583 (30.9%)	0.027
Pharmacy	352 (22.5%)	8 (4.4%)	19 (16.7%)	2 (7.4%)	381 (20.2%)	< 0.001
African healer	40 (2.6%)	2 (1.1%)	1 (0.9%)	1 (3.7%)	44 (2.3%)	0.422
Friends	92 (5.9%)	21 (11.7%)	20 (17.5%)	5 (18.5%)	138 (7.3%)	< 0.001
I do not knowwhere to go	3 (0.2%)	2 (1.1%)	18 (15.8%)	3 (11.1%)	26 (1.4%)	< 0.001
Other	35 (2.2%)	8 (4.4%)	16 (14.0%)	0 (0.0%)	59 (3.1%)	< 0.001

Multiple answers were possible

### Last consultation for medical care

In total 44% of participants consulted a physician or a hospital within the past month, an additional 35% consulted them in the past 12 months. Every tenth (9.9%) reported a consultation within the last five years; for 33 participants (1.7%) it was more than five years ago and 8.5% could not remember their last consultation.

To analyse the impact of health insurance status on the last consultation for medical care, we used a MVA (if there was a medical consultation within the last year). Female individuals compared to males (aOR = 1.90; 95% CI: 1.44–2.5) and older participants (≥ 46 years) compared to younger participants (26–35 years) (aOR = 1.87; 95% CI: 1.17–2.99), were consulting a physician or hospital more often. Participants with no degree or certificate compared to those with a primary or secondary school degree (aOR = 0.57; 95% CI: 0.34–0.97) and participants who had NI compared to HIC (aOR = 0.36; 95% CI: 0.21–0.60), were consulting a physician or hospital less often. No differences were detected between participants with HIC and MTV (Table 6).

**Table 6:** Multivariate analysis on the last consultation of a physician or hospital (within one year = 1, over one year ago = 0), *n* = 1574

Variable	%	Univariate Analysis			Multivariate Analysis		
		OR	95% - CI	*p*-value	aOR	95% - CI	*p*-value
Sex							
Men	75.2%	Ref.			Ref.		
Women	84.9%	**1.86**	**1.47–2.35**	**< 0.001**	**1.90**	**1.44–2.50**	**< 0.001**
Age group							
18–25 years	76.3%	0.98	0.72–1.34	0.900	1.12	0.77–1.62	0.567
26–35 years	76.7%	Ref.			Ref.		
36–45 years	82.1%	**1.40**	**1.00–1.90**	**0.032**	1.21	0.85–1.72	0.293
≥ 46 years	85.0%	**1.72**	**1.22–2.43**	**0.002**	**1.87**	**1.17–2.99**	**0.009**
Length of stay in Germany							
≤ 1 year	69.7%	**0.57**	**0.38–0.85**	**0.006**	0.66	0.36–1.20	0.170
1 - < 5 years	77.5%	0.85	0.61–1.18	0.340	0.92	0.61–1.38	0.681
5 - < 10 years	80.2%	Ref.			Ref.		
10 - < 20 years	83.3%	1.23	0.86–1.75	0.251	1.08	0.72–1.64	0.707
≥ 20 years	83.8%	1.28	0.84–1.95	0.244	0.96	0.55–1.65	0.869
German language skills							
None	73.6%	0.72	0.47–1.10	0.127	1.76	0.91–3.42	0.096
Little	76.9%	0.86	0.61–1.19	0.353	1.14	0.75–1.75	0.541
Satisfactory	82.1%	1.18	0.85–1.65	0.323	1.46	0.99–2.15	0.058
Good	79.6%	Ref.			Ref.		
Very good	80.8%	1.08	0.78–1.49	0.639	1.01	0.76–1.59	0.628
Mother tongue	81.0%	1.09	0.49–2.43	0.829	1.02	0.42–2.48	0.973
Religion							
Christian	80.5%	Ref.			Ref.		
Muslim	79.2%	0.92	0.71–1.21	0.559	1.17	0.85–1.60	0.338
No or other religion	73.9%	0.69	0.44–1.07	0.092	0.82	0.49–1.34	0.421
Education							
Other	76.5%	0.71	0.23–2.27	0.553	0.78	0.21–2.92	0.710
No degree or certificate	74.6%	0.64	0.40–1.02	0.059	**0.57** **00.54**	**0.34–0.97**	**0.039**
Primary or secondary school	82.1%	Ref.			Ref.		
High −/vocational school	79.0%	0.82	0.61–1.11	0.200	0.78	0.54–1.11	0.167
University degree	79.4%	0.84	0.62–1.16	0.255	0.85	0.59–1.24	0.399
Monthly NET income							
I do not want to answer	76.8%	0.86	0.60–1.22	0.396	1.09	0.73–1.63	0.675
No income	75.6%	**0.80**	**0.55–1.17**	**0.250**	1.05	0.67–1.67	0.821
< 500 €	78.0%	0.92	0.62–1.36	0.666	1.11	0.70–1.75	0.661
500 € - < 1.000 €	79.4%	Ref.			Ref.		
1.000 € - < 2.000 €	82.0%	1.18	0.83–1.69	0.353	1.33	0.89–2.00	0.167
≥ 2.000 €	87.1%	1.74	0.96–3.18	0.067	1.89	0.98–3.65	0.059
Health insurance status							
Health insurance card	81.2%	Ref.			Ref.		
Medical treatment voucher	79.4%	0.90	0.61–1.32	0.578	1.03	0.62–1.71	0.905
No insurance	60.5%	**0.36**	**0.24–0.53**	**< 0.001**	**0.36**	**0.21–0.60**	**< 0.001**

Significant results are marked bold

Duration of residence in Germany, German language skills, religion and income were not associated with the reported last consultation with a physician or hospital.

### Uptake of HIV testing

Two thirds of the participants reported having ever been tested for HIV (66%), 30% had never been tested and 4.4% did not know.

In MVA, female gender (aOR = 1.98; 95% CI: 1.24–2.05) and having a university degree (aOR = 1.81; 95% CI: 1.29–2.54) or high−/vocational school degree (aOR = 1.44; 95% CI: 1.05–1.97) were associated with a higher uptake of HIV testing. Also participants of older age (≥36 years) (36–45 years aOR = 2.03; 95% CI: 1.43–2.88; ≥ 46 years; aOR = 1.85; 95% CI: 1.21–2.82), with a monthly net income of 2000 € or more (aOR = 2.17; 95% CI: 1.16–4.10) and those originating from Central (aOR = 1.48; 95% CI: 1.08–2.01) or Eastern Africa (aOR = 1.57; 95% CI: 1.08–2.27) had higher odds of ever having had an HIV test. Participants with Muslim religion (aOR = 0.70; 95% CI: 0.52–0.93), with no school degree or certificate (aOR = 0.49; 95% CI: 0.31–0.79), with a long duration of residence in Germany (≥ 20 years; aOR = 0.59; 95% CI: 0.36–0.98), younger participants (18–24 years; aOR = 0.42; 95% CI: 0.30–0.56) and those who had done a face-to-face (aOR = 0.71; 95% CI: 0.55–0.91) or telephone interview (aOR = 0.53; 95% CI: 0.35–0.78) had less often ever been tested for HIV. Participants with NI less often had been tested for HIV than those with HIC (aOR = 0.55; 95% CI: 0.31–0.95) (Table 7).

**Table 7:** Multivariate analysis on HIV test uptake (ever been tested = 1, not tested = 0), *n* = 1514

Variable	%	Univariate Analysis			Multivariate Analysis		
		OR	95% - CI	*p*-value	aOR	95% - CI	*p*-value
Sex							
Men	62.2%	Ref.			Ref.		
Women	70.4%	**1.44**	**1.19–1.75**	**< 0.001**	**1.59**	**1.24–2.05**	**< 0.001**
Age group							
18–25 years	42.1%	**0.36**	**0.27–0.47**	**< 0.001**	**0.42**	**0.30–0.56**	**< 0.001**
26–35 years	67.0%	Ref.			Ref.		
36–45 years	77.8%	**1.72**	**1.30–2.28**	**< 0.001**	**2.03**	**1.43–2.88**	**< 0.001**
≥ 46 years	70.2%	1.16	0.87–1.53	0.308	**1.85**	**1.21–2.82**	**0.005**
Length of stay in Germany							
≤ 1 year	55.7%	**0.56**	**0.39–0.80**	**0.002**	1.82	1.00–3.33	0.051
1 - < 5 years	61.7%	**0.72**	**0.54–0.95**	**0.021**	1.05	0.71–1.55	0.808
5 - < 10 years	69.3%	Ref.			Ref.		
10 - < 20 years	72.8%	1.19	0.88–1.61	0.267	0.84	0.57–1.24	0.381
≥ 20 years	66.2%	0.87	0.62–1.22	0.417	**0.59**	**0.36–0.98**	**0.042**
German language skills							
None	54.7%	**0.66**	**0.46–0.96**	**0.029**	0.98	0.53–1.82	0.951
Little	61.7%	0.87	0.67–1.18	0.407	1.27	0.85–1.91	0.249
Satisfactory	70.2%	1.29	0.98–1.71	0.073	1.42	1.00–2.01	0.050
Good	64.5%	Ref.			Ref.		
Very good	70.2%	1.30	0.98–1.71	0.067	1.16	0.82–1.63	0.405
Mother tongue	66.7%	1.10	0.56–2.14	0.783	1.67	0.70–3.99	0.247
Religion							
Christian	70.3%	Ref.			Ref.		
Muslim	52.2%	**0.46**	**0.37–0.58**	**< 0.001**	**0.70**	**0.52–0.93**	**0.014**
No or other religion	72.2%	1.10	0.72–1.68	**0.665**	1.28	0.77–2.13	0.338
Education							
Other	58.8%	1.00	0.38–2.68	0.993	0.52	0.15–1.75	0.288
No degree or certificate	43.9%	**0.55**	**0.37–0.82**	**0.003**	**0.49** **00.54**	**0.31–0.79**	**0.003**
Primary or secondary school	58.7%	Ref.			Ref.		
High −/vocational school	66.8%	**1.42**	**1.10–1.82**	**0.006**	**1.44**	**1.05–1.97**	**0.025**
University degree	74.7%	**2.07**	**1.61–2.68**	**< 0.001**	**1.81**	**1.29–2.54**	**0.001**
Monthly NET income							
I do not want to answer	68.5%	1.10	0.80–1.51	0.559	1.38	0.95–2.01	0.096
No income	49.4%	**0.49**	**0.36–0.68**	**< 0.001**	0.68	0.45–1.02	0.061
< 500 €	63.5%	0.88	0.62–1.24	0.458	1.17	0.77–1.78	0.462
500 € - < 1.000 €	66.5%	Ref.			Ref.		
1.000 € - < 2.000 €	72.1%	1.31	0.96–1.77	0.086	1.34	0.93–1.94	0.115
≥ 2.000 €	83.9%	**2.63**	**1.52–4.57**	**< 0.001**	**2.17**	**1.16–4.10**	**0.016**
Health insurance status							
Health insurance card	68.0%	Ref.			Ref.		
Medical treatment voucher	58.7%	**0.67**	**0.49–0.92**	**0.012**	1.11	0.70–1.78	0.655
No insurance	52.7%	**0.52**	**0.36–0.77**	**0.001**	**0.55**	**0.31–0.95**	**0.033**
Region of birth							
Western Africa	60.4%	Ref.			Ref.		
Central Africa	72.2%	**1.70**	**1.33–2.17**	**< 0.001**	**1.48**	**1.08–2.01**	**0.014**
Eastern Africa	73.7%	**1.83**	**1.35–2.50**	**< 0.001**	**1.57**	**1.08–2.27**	**0.018**
Southern Africa	73.3%	**1.79**	**1.14–2.84**	**0.011**	1.28	0.75–2.17	0.371
Administration mode							
Face-to-face	62.1%	**0.65**	**0.53–0.79**	**< 0.001**	**0.71**	**0.55–0.91**	**0.008**
Self-administered	71.7%	Ref.			Ref.		
Telephone	59.9%	**0.59**	**0.42–0.83**	**0.002**	**0.53**	**0.35–0.78**	**0.001**

Significant results are marked bold

## Discussion

Health insurance status was mainly associated with the length of stay in Germany and German language skills. NI participants less often used health care, if they had health problems than participants with HIC or MTV. At the same time participants with NI were significantly less likely to be tested for HIV.

### Access to health care

Longer duration in Germany and higher levels of German language skills were significantly associated with a larger proportion of participants having health insurance. Thus, it seems that differences in health insurance status are not persisting over time but mainly observed within the first years after migration. The proportion of those with restricted access to health care through a MTV was especially high within the first five years. As described, migrants in Germany have the right to access the regular health care system after 15 months of stay. However, there were 91 participants who lived in Germany for more than 15 months and still accessed medical services through a MTV. These cases indicate that the law is not observed in all cases.

Several studies have shown that migrants have restricted access to the health care system compared to the general population. Reasons include lack of legal residence status, fear of discrimination or deportation, stigma and less knowledge about the health system [7, 9, 10]. As studies showed, political solutions are necessary to ensure access to health care and sufficient preventive and medical care for everybody [24, 25].

In our findings, lack of German language skills and lower educational status were associated with a MTV or NI. On the one hand, misSA that migrate to Germany to study are entitled to the compulsory health insurance. On the other hand, misSA with a higher education and better German language skills might have fewer difficulties obtaining the necessary information on integration, residence, insurance and health care system [14–16] which can expedite the integration into the standard health care system. This underlines the necessary language support for migrants, especially within the first years of residence in Germany. German language skills may facilitate misSA to obtain information on the health system and assert their rights. Still, information in multiple languages should be made accessible to enable culturally sensitive services for those who are not yet fluent in German. We also noticed that 12 out of 27 participants, who did not know whether they had health insurance or not, reported not speaking German. This also emphasizes the need for multilingual information access. However, it is likely that other factors related to the rights of residence (like a refused asylum request), are more important components than socio-demographic characteristics, to explain an irregular insurance status [9, 10, 14, 15]. We do not know to what extent this played a role since this sensitive information was not collected from the participants.

### Utilization of services

We did not detect differences in utilization of physicians or hospitals between participants with HIC and MTV. This shows that the basic care for this population seems to work well.

Females were significantly more often consulting physicians or hospitals, which most presumably is a result of gender-specific differences in health seeking behavior [2]. However, we observed that especially those with NI had a lower utilization of health care, independently of other socio-demographic factors. It seems that compared to those with MTV even basic care is not granted for this group.

If a person has neither HIC nor MTV and cannot pay medical bills individually, those bills are sent to the social welfare office and thereby personal data of this person is forwarded to the foreigners’ registration office. Without a residence permit this might lead to deportation or persecution. This potential risk leads to enormous psychological stress and might result in late presentation and in complications of initially treatable conditions [15, 16]. Nevertheless, 60% of participants with NI reported consulting a physician within the past year. This points towards a functional network among irregular migrants and medical care services outside of the regular system, for example offices for medical assistance for refugees (Medibueros) [11].

### Uptake of HIV testing

Diverse socio-demographic factors were identified in the analysis that determined the uptake of HIV testing.

Female participants had a higher uptake of HIV testing than men. Most female participants were of reproductive age and the HIV test is a standard offer of antenatal care for pregnant woman in Germany [26]. Also for older participants the odds of having a HIV test increased. As studies showed, the odds of older adults of being tested are higher compared to younger adults, because they have had more opportunities for testing over their lifespan [26, 27]. Muslim participants had a significantly lower uptake of HIV testing than Christians or participants with no or other religion. This is perhaps because sexuality and HIV are bigger taboos in Muslim communities than in the other groups [27].

A higher school degree increased the odds of having been tested for HIV. As described in other publications, people with higher educational levels are more aware of the risk of HIV, know more about testing offers and are more easily reached by prevention [26].

Participants who did a face-to-face or telephone interview reported less often to be tested for HIV than participants that filled the questionnaire out by themselves. This may be because HIV is still a taboo and it might be difficult to talk about it or to admit taking an HIV test when doing the interview with somebody from their own African community.

Participants with NI had lower odds of ever being tested for HIV. A study in the UK showed that also an uncertain residence status is one of the main concerns of African migrants and can be a deterrent for HIV testing and accessing services [28].

Another barrier which might lead to a lower HIV test uptake is the lack of treatment options for migrants with NI or only restricted access to HIV medication for migrants with MTV in Germany. To counteract this, political decisions are necessary like in other European countries, e.g. in the UK, where HIV treatment is included in the National Health Service [29]. The information, that there exist free and anonymous HIV testing services in Germany should be spread out and could lead to a higher uptake of HIV testing as well.

### Limitations

There are some limitations to consider when interpreting these results. To reach misSA in different living conditions, convenience sampling was chosen as a useful method of recruitment. To map large communities in Germany, statistics of German foreigner’s registration offices has been used. These include nationalities and sex ratio, which means that other socio-demographic factors of the misSA population in Germany are not known. Additionally, there is no specific information about non-registered misSA, misSA with German citizenship as well as misSA without health insurance living in Germany. Hence, representativeness cannot be ensured due to uncertainty about the sampling frame and the sampling method. The conditions for a random sample for statistical testing and measurement are eventually not fulfilled and ratios and expected ranges in the population are rather to be perceived as a tendency.

The educational level of the study population is higher than the German average. There is no reliable information on educational degrees of misSA who live in Germany and an educational bias cannot be excluded, but peer researchers from Berlin reported that there are many misSA who come to Germany for university studies [30].

Furthermore, a recall bias, due to events occurring a long time ago as well as a bias due to social desirability, especially in personal interviews is possible. We tested the significances of survey administration and it showed no impact except for the uptake of HIV testing.

The questionnaire was offered in English, French and German but peer researchers had knowledge about many African languages. However, the survey was not offered in any African language and misSA who could not read any of the presented languages and did not want to do a personal interview might be underrepresented, which may have led to a selection bias.

## Conclusion

We were able to show that the absence of regular health insurance by misSA increased the odds of no contact with the healthcare system in the past year more than other socio-demographic characteristic. Furthermore, misSA without health insurance were less likely to have been tested for HIV than insured participants. To increase utilization of (preventive) health care and testing services and to ensure adequate medical care, everybody should obtain access to health insurance. To ensure universal access to health care as a human right, political solutions are necessary.

Multilingual services should be offered in order to inform recent migrants and individuals with low German language skills properly on residence status and the German health system and to ensure cultural sensitivity.

To reach a higher uptake of HIV testing education and prevention programmes should target specifically particularly vulnerable subgroups such as males, people less than 26 years of age, misSA with lower school education as well as migrants without health insurance.

## Abbreviations

BZgA: Federal Centre for Health Education (Bundeszentrale für gesundheitliche Aufklärung)
DAH: German AIDS Service Organization (Deutsche Aids-Hilfe)
ECDC: European Centre for Disease Prevention and Control
HEP: Viral hepatitis
HIC: Health insurance card
HIV: Human immunodeficiency virus
KABP: Survey on knowledge, attitude, behavior, practice
MisSA: Migrants from sub-Saharan Africa
MTV: Medical treatment voucher
MVA: Multivariate analysis
NGO: Non-governmental organization
NI: Migrants without health insurance
OR: Odds Ratios
RKI: Robert Koch Institute
STI: Sexually transmitted infections
WHO: World Health Organization
